# Fuzzy Diagnostic System for Oleo-Pneumatic Drive Mechanism of High-Voltage Circuit Breakers

**DOI:** 10.1155/2013/248487

**Published:** 2013-11-04

**Authors:** Viorel Nicolau

**Affiliations:** “Dunarea de Jos” University of Galati, 47 Domneasca Street, 800008 Galati, Romania

## Abstract

Many oil-based high-voltage circuit breakers are still in use in national power networks of developing countries, like those in Eastern Europe. Changing these breakers with new more reliable ones is not an easy task, due to their implementing costs. The acting device, called oleo-pneumatic mechanism (MOP), presents the highest fault rate from all components of circuit breaker. Therefore, online predictive diagnosis and early detection of the MOP fault tendencies are very important for their good functioning state. In this paper, fuzzy logic approach is used for the diagnosis of MOP-type drive mechanisms. Expert rules are generated to estimate the MOP functioning state, and a fuzzy system is proposed for predictive diagnosis. The fuzzy inputs give information about the number of starts and time of functioning per hour, in terms of short-term components, and their mean values. Several fuzzy systems were generated, using different sets of membership functions and rule bases, and their output performances are studied. Simulation results are presented based on an input data set, which contains hourly records of operating points for a time horizon of five years. The fuzzy systems work well, making an early detection of the MOP fault tendencies.

## 1. Introduction

In power networks, the quality and security problems are correlated with each other, being extensively debated in the literature. The increased concern for power quality has resulted in significant advances of monitoring and diagnostic techniques that can be used to characterize disturbances and power quality variations [1]. Different analysis methods can point out individual events, statistical summaries, or trends.

The circuit breakers (CB) play an important role for network stability and security, as for power quality. Knowing the actual and future states of circuit breakers is one of the primary requisites to achieve the goals of optimal planning and operation of the large-scale power networks. As most components of power networks, CB should be enabled to detect their potential failures and make life expectancy prediction without human interference [2]. Diagnostic and monitoring techniques can be used to optimize maintenance practices, replacement, and utilization of circuit breakers. But these procedures must be selected with realistic expectations, as discussed [3].

Changing the old circuit breakers with new ones is not an easy task, even in developed countries. The cost of the breaker and station equipment becomes prohibitive at high voltages [4]. In addition, their huge number in the power networks makes the renewing process impossible in a short period of time. Hence, for aged circuit breakers, a solution could be new monitoring and diagnostic intelligent systems.

Monitoring of the main functions of circuit breakers assures less expensive exploitation during its life by less regular maintenance [5]. Also, it improves the overall performances, like reliability and availability of the large-scale power networks [6]. At the same time, the monitoring and diagnostic procedures represent main solutions to prevent failures, safely decrease the scheduled maintenance costs, and minimize downtime [7]. A development of diagnostic and monitoring system for circuit breakers is presented in [8].

The state estimation is a key function for building a circuit breaker real-time model. In [9], an overview of electric power system state estimation is presented, starting from conventional state estimation, where logical and analog data are studied separately. Also, probabilistic assessment methods can be used to express the reliability of power systems, and in particular of high-voltage circuit breakers. In [10], probabilistic criteria and indexes are presented and some new areas of development are pointed-out.

In national power networks of developing countries, there are still many IO-type oil-based high voltage circuit breakers, which are operated by oleo-pneumatic mechanisms (MOP). The circuit breakers are aged, many of them having overpassed their life time, as discussed in [11]. Due to their low reliability, it is important to implement new less-expensive monitoring and diagnostic equipments for the major parts of IO-type circuit breakers. They should use advanced prediction techniques to detect their failure trends and make life-expectancy estimation.

The behavior analysis of IO-type circuit breakers pointed out that, from all breaker components, the oleo-pneumatic drive mechanism presents the highest fault rate [11]. Its failure goes to the impossibility of switching the CB, which may affect the power network stability and security, and also the power quality. Therefore, knowing the actual and future states of MOP-type mechanisms is important to achieve the goals of optimal planning and operation of the power networks. Modern methods for off-line diagnosis of MOP-type mechanisms are presented in [11].

There are many papers in the literature, using the artificial intelligence methods to decrease the exploitation costs and to improve the overall performances of high voltage circuit breakers from power networks. The basic principles for prediction and condition diagnosis from the field of artificial intelligence are stated in [12].

Fuzzy logic has proved its efficiency in monitoring and diagnosis of different components of power networks. A fuzzy diagnostic system for estimating the fault section of power system is presented in [13], and an adaptive version using matrix representations with fuzzy relations is introduced in [14]. Online fault diagnosis system on a transmission network can be implemented based on fuzzy Petri nets [15] or with a fuzzy expert system, as shown in [16]. An adaptive fuzzy system was used in [17] for learning power-quality signature waveforms. Also, neurofuzzy techniques were used together in different power system applications, such as in [18] for predictive maintenance with self-diagnosis capability at the substation equipment level. A neurofuzzy system for state estimation and fault detection in power systems was proposed in [19].

In this paper, fuzzy logic approach is used for early diagnosis of MOP-type drive mechanisms. A fuzzy system is proposed for online predictive diagnosis and early detection of the fault tendencies of MOP mechanisms.

The paper is organized as follows.  Section 2 presents diagnostic aspects of MOP-type drive mechanisms. Expert rules for predictive diagnosis are generated in Section 3. Fuzzy diagnostic system is studied in Section 4 and simulation results are presented in Section 5. Conclusions are pointed out in Section 6.

## 2. Diagnostic Aspects of MOP-Type Mechanism

The IO-type high-voltage circuit breakers are operated by oleo-pneumatic drive mechanisms. The acting energy is stored into an accumulator with nitrogen under pressure. From there, when needed, the energy is transmitted to mobile contacts of circuit-breaker through a hydraulic system. The nitrogen pressure must be high enough to trigger the switching process. 

The main drawback of this acting mechanism is the accumulator, which loses in time the internal pressure, meaning that it loses the stored energy. When this is detected, a pump actuated by a motor restores the pressure. The pressure variation is detected through a piston displacement, using two on-off microswitches. Their positions correspond to maximum and minimum acceptable values of internal nitrogen pressure. Within this range, the internal pressure is considered as normal.

Pressure loss is a natural process and it cannot be avoided. In normal operation, the motor starts several times every hour. The number of starts per hour (*N*_*S*_) can be a malfunction indicator of pressure recovery circuit. If this number is too big, this means the accumulator loses the internal pressure too quickly and the recovery circuit is working too often. On the contrary, if the motor does not start during one hour, this can indicate a malfunction of pressure variation sensing.

In addition, the total functioning time per hour (*T*_*F*_) can also indicate a malfunction. In normal operation, the motor is functioning for a few seconds for every start. If the motor is functioning for long time periods every hour, this means that the pressure is restored too slowly due to a failure in the pump-motor circuit.

The number of starts and total functioning time per hour of the pump-motor group differ from one mechanism to another, depending on internal and external disturbances. The internal disturbances (*w*_*i*_) refer to wear and tear of various components, such as valves and electrovalves. The external ones (*w*_*e*_) refer to weather conditions, especially temperature which affects the high-pressure oil circuits. As a result, different MOP mechanisms can have normal behaviors, although their pump-motor groups have different values for the number of starts and total functioning time per hour.

Regarding normal operation of pump-motor group, every MOP mechanism must fulfill two performance conditions as follows.

(i) The number of starts per hour (*N*_*S*_) changes in time, depending on *w*_*i*_ and *w*_*e*_. It must be within a predefined value range, specified in technical datasheet. This means that the motor must start at least *N*_*S*min⁡_ times in an hour, but not too often, no more than *N*_*S*max⁡_ as follows:
  (1) $$\begin{array}{c} N_{S}\left(t,w_{i},w_{e}\right)\in \left[N_{S\min},N_{S\max}\right]. \end{array}$$
(ii) The total functioning time of the motor into an hour (*T*_*F*_) must not exceed a maximum value, denoted *T*_*F*max⁡_, also found in technical specifications. The minimum value, *T*_*F*min⁡_, is not specified, but it is nonzero value, being correlated with *N*_*S*min⁡_ as follows:
  (2) $$\begin{array}{c} T_{F}\left(t,w_{i},w_{e}\right)\in \left[T_{F\min},T_{F\max}\right]. \end{array}$$

The normal operation point of MOP-type drive mechanism, denoted *P*, can be characterized in *ℜ* ^2^ by a pair of coordinates changing in time, (*x*_1_(*t*), *x*_2_(*t*)) ∈ *ℜ* ^2^ as follows:

(3) $$\begin{array}{c} x_{1}\left(t\right)=N_{S}\left(t,w_{i},w_{e}\right),\;\;x_{2}\left(t\right)=T_{F}\left(t,w_{i},w_{e}\right), \end{array}$$

with restrictions defined by performance conditions in (1) and (2), respectively.

**Figure 1:**
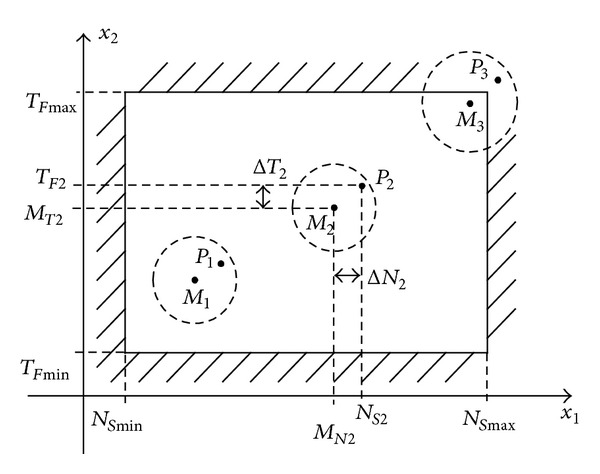
Variations of normal operating point *P* in *ℜ* ^2^ within the permitted area.

In normal operation, due to internal and external disturbances, the point *P* is shifting in *ℜ* ^2^ within the permitted area, as shown in Figure 1. The restriction boundaries are illustrated with a shaded area.

Considering their frequency characteristics, perturbations can be divided into 3 categories: low-, medium-, or high-frequency, denoted *w*_*l*_, *w*_*m*_, and *w*_*h*_, respectively. They can be considered as additive perturbations for the model of MOP-type mechanism. Accordingly, there are 3 different influences on the MOP operating point, depending on the time horizon which is considered long-, medium-, or short-term time period as follows:

(4) $$\begin{array}{c} x_{1}\left(t\right)=x_{1S}\left(t,w_{h}\right)+x_{1M}\left(t,w_{m}\right)+x_{1L}\left(t,w_{l}\right),\\ x_{2}\left(t\right)=x_{2S}\left(t,w_{h}\right)+x_{2M}\left(t,w_{m}\right)+x_{2L}\left(t,w_{l}\right), \end{array}$$

where *x*_*iS*_, *x*_*iM*_, or *x*_*iL*_ are the short-, medium-, or long-term components of *i* coordinate, *i* = 1, 2.

The time interval length depends on the process type. For oleo-pneumatic drive mechanisms, short-term refers to days and weeks, medium extends to months or one year, and long-term takes into account several years to the life cycle.

Short-term perturbations are of random nature and produce variations of *P* point around the mean value, denoted *M*, computed over a predefined time horizon. The mean value includes medium- and long-term components, and it is also varying in time. As the time horizon is higher, the variations of mean value will be smaller.

In Figure 1, the boundaries of short-term random variations are represented as circles with dashed lines around mean values. The point coordinates can be rewritten as follows:

(5) $$\begin{array}{c} x_{1}\left(t\right)=M_{N}\left(t\right)+\Delta N\left(t\right),\\ x_{2}\left(t\right)=M_{T}\left(t\right)+\Delta T\left(t\right), \end{array}$$

where Δ*N*(*t*) and Δ*T*(*t*) are the short-term components, considered as random variables with normal distribution and zero mean. The mean values, *M*_*N*_(*t*) and *M*_*T*_(*t*), are functions of time *t*, including medium- and long-term components from (4), *x*_1*M*_, *x*_2*M*_, and *x*_1*L*_, *x*_2*L*_, respectively.

## 3. Expert Rules for Predictive Diagnosis of MOP Functioning

In this section, expert rules are generated to characterize the functioning state of MOP-type drive mechanism. The MOP behavior analysis is based on variations in the time of medium- and long-term components of mean values from (5).

On medium-term, during one year period, the mean value of normal operating point has a cyclic movement, as the temperature is changing from one season to another. 

**Figure 2:**
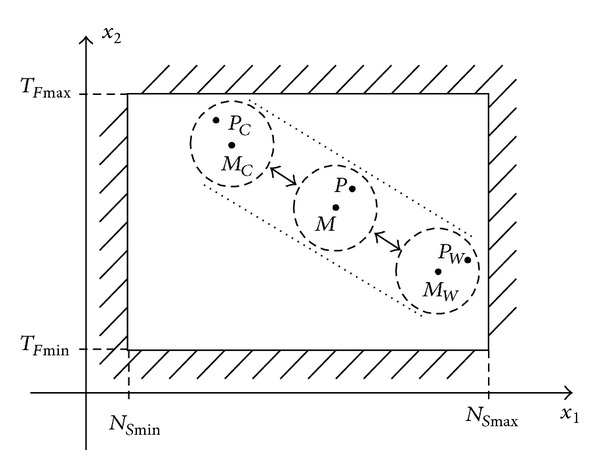
Cyclic movement of mean value of normal operation point on medium-term.

In cold periods, due to low temperatures, the oil has higher viscosity and mechanical friction increases. The pump-motor group must work for longer time periods to restore the pressure. As a result, the number of starts per hour decreases and total functioning time per hour increases. The normal operating point and its mean value, denoted in this case *P*_*C*_ and *M*_*C*_, are moving to the upper-left corner of the permitted area, as shown in Figure 2. The boundaries of short-term random variations are marked as dashed circles, and the uncertainty tunnel of cyclic movement is represented with dotted lines.

*Rule No. 1.* In cold periods, shifting the operating point and its mean value to upper-left corner of permitted area can be a normal process. It is not necessarily a defective functioning state.

On the contrary, in warm periods, the oil has low viscosity. Hence, the number of starts increases, and total functioning time decreases. The operating point and its mean value, denoted *P*_*W*_ and *M*_*W*_, are moving to the lower-right corner of the permitted area. 

*Rule No. 2.* In warm periods, the operating point and its mean value can move to the lower-right corner of permitted area as a normal process, and this is not necessarily a defective functioning state.

During life cycle, the aging process of circuit breakers leads to worsening performance. As a result, parameters, number of starts, and functioning time, increase. The mean value of operating point is slowly changing from the lower-left to the upper-right corner, as illustrated in Figure 1 for three different situations. In addition, the variations of *P* point on short- and medium-term can increase. 

*Rule No. 3.* Due to the aging process of circuit breakers, on high horizon of time, the operating point is shifting to the upper-right corner of the permitted area.

**Figure 3:**
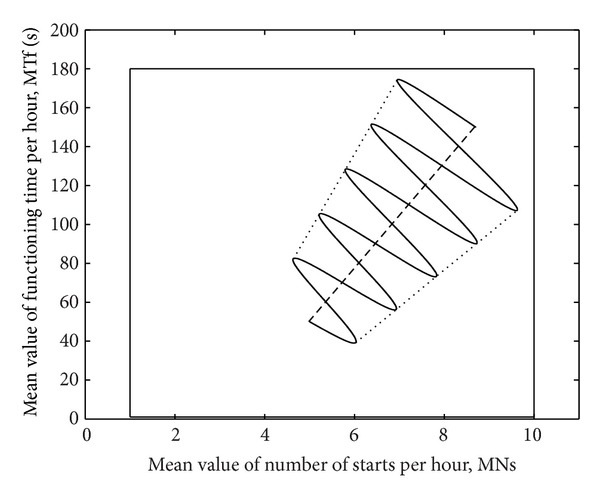
Variations in the time of mean values including medium- and long-term components.

The mean values from (5), *M*_*N*_(*t*), and *M*_*T*_(*t*), are functions of time *t* and include medium- and long-term components. Their time variations are shown for an example in Figure 3.

The variation of long-term mean value is considered as linear, being represented with a dashed line. For cyclic movement of medium-term mean value, a smooth periodic function like a sinus is considered, with increasing magnitude in time.

The uncertainty tunnel of mean value variations is represented with dotted lines in Figure 3. By combining the medium- and long-term variations of mean values, the resulting trajectory (continuous line) is sinusoidal, also with increasing magnitude.

As the mean value of the operating point reaches the upper-right corner in the permitted area, short-term perturbations can determine the operating point to cross the restriction boundaries. Such an example, corresponding to *P*_3_ and *M*_3_ situation, is illustrated in Figure 1. Thus, performance conditions from (1) and (2) are not met, even if the MOP is not defective. In addition, the uncertainty region delimited by the boundaries of random variations increases, so the circle drawn with a dashed line is bigger. 

*Rule No. 4.* If the mean values are near the border of permitted area, the operating point can cross the border for short periods of time.

**Figure 4:**
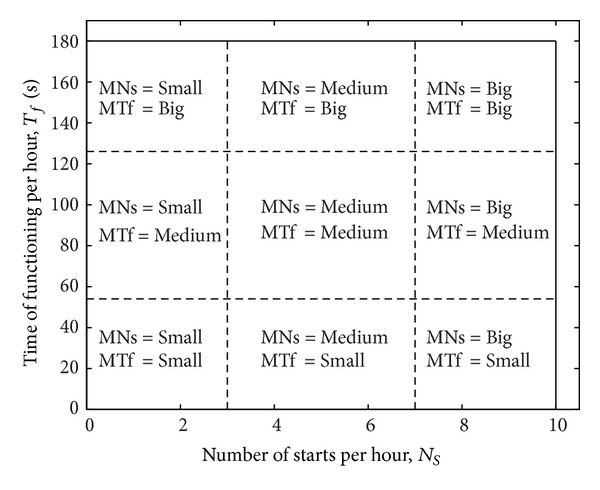
Zones generated in the permitted area by fuzzy inputs *Mean-NS* and *Mean-TF.*

The mean values from (5), *M*_*N*_(*t*) and *M*_*T*_(*t*) can be used as fuzzy variables, denoted *MNs*, and *MTf,* with three membership functions called: *Small*, *Medium*, and *Big*. By combining their membership functions, these two fuzzy variables divide the permitted area in *ℜ* ^2^ into 9 different zones, as shown in Figure 4.

In every zone, the pair of mean values characterizes the functioning state of the MOP in conjunction with short-term components from (5), Δ*N*(*t*), and Δ*T*(*t*).

There are zones, for which the functioning state can be specified based on the pair of mean values alone.

*Rule No. 5.* If the MNs are Small and MTf is Small, then the MOP state is Good.

*Rule No. 6.* If the MNs are Medium and MTf is Medium, then the MOP state is Normal. 

*Rule No. 7.* If the MNs are Big and MTf is Big, then the MOP state is Bad. 

By refining the MOP functioning state (e.g., Good and Very good) the rules must include the short-term components as fuzzy variables.

## 4. Fuzzy Diagnostic System

Fuzzy logic can be used to estimate the functioning state of MOP-type mechanisms, on a scale from 0 to 10, taking into account the behavior aspects near the boundaries of permitted area. In this way, online predictive diagnosis and early detection of the fault tendencies can be accomplished.

The fuzzy information is useful in the programming of major overhauls and for decreasing the mean values of the operating point. Also, the fuzzy system avoids the unnecessary functioning interrupts, like in crisp diagnostic system, when the MOP is not yet defective, even if the performance conditions are not met for short periods of time.

**Figure 5:**
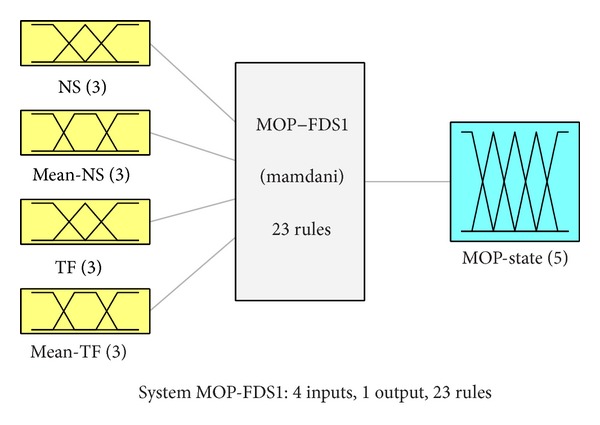
Structure of fuzzy diagnostic system.

In this section, a fuzzy diagnostic system (FDS) is proposed to characterize the functioning state of MOP-type mechanism, based on the model expressed in (5). It is a Mamdani-type fuzzy system, denoted MOP-FDS, with 4 inputs and 1 output. The FDS structure is shown in Figure 5.

Different fuzzy systems were studied in simulations, denoted MOP-FDSi, with *i* = 1,2, 3,…, with different characteristics of knowledge base and membership functions.

**Figure 6:**
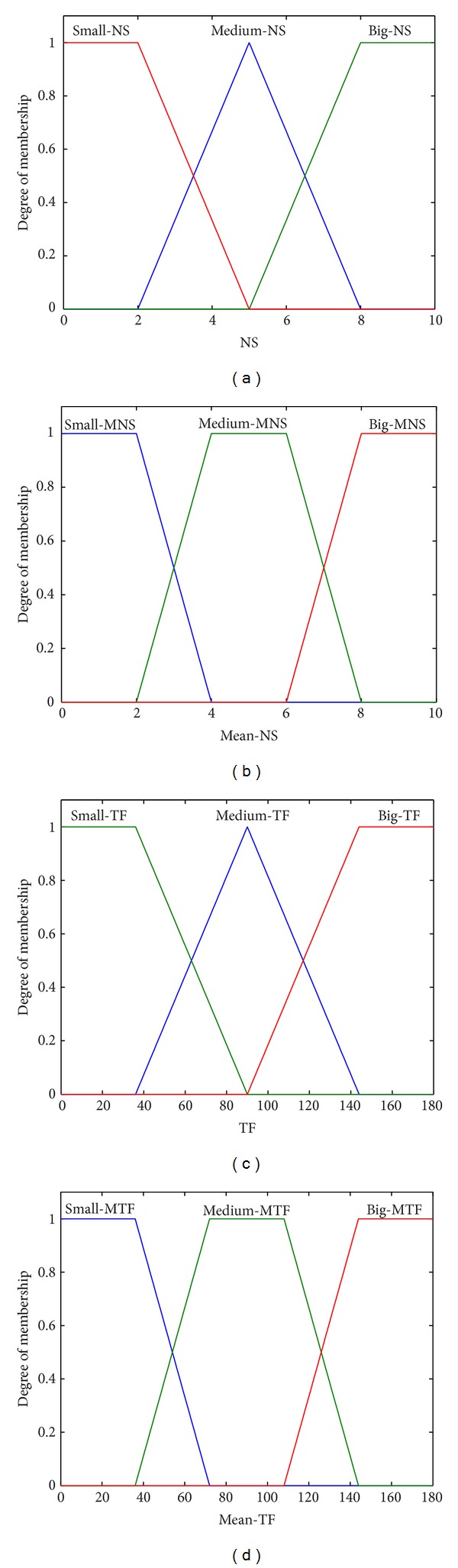
Membership functions of FDS inputs.

The inputs give information about the number of starts and time of functioning per hour, in terms of short-term components, *NS* and *TF*, and their mean values from (5), *Mean-NS* and *Mean-TF*, respectively. An example is illustrated in Figure 6. 

The ranges of membership functions are chosen based on restrictions expressed in (1) and (2), with limited values from technical specifications.

Each input of MOP-FDS has three membership functions, denoted: Small (S), Medium (M), and Big (B). The first two fuzzy inputs characterize the number of starts per hour. The *NS* fuzzy variable has membership functions denoted: *Small-NS, Medium-NS, *and* Big-NS*. The membership functions for *Mean-NS* fuzzy variable are: *Small-MNS, Medium-MNS, *and* Big-MNS*. Similarly, the other two inputs for time of functioning per hour, *TF* and *Mean-TF*, have the membership functions denoted: *Small-TF, Medium-TF, *and* Big-TF*, and *Small-MTF, Medium-MTF, *and* Big-MTF*, respectively.

**Figure 7:**
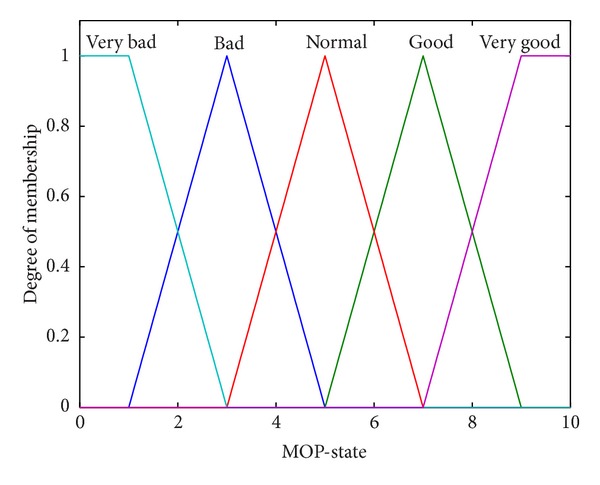
Membership functions of FDS output.

The fuzzy output (*MOP-State*) estimates the functioning state of MOP-type mechanism, on a scale from 0 to 10. It has 5 membership functions, represented in Figure 7.

The output membership functions are denoted: *Very Bad* (*VB*), *Bad* (*B*), *Normal* (*N*), *Good* (*G*), and *Very Good* (*VG*).

The complete rule base has 81 simple fuzzy rules, but it can be generated with much less but more complex rules. An example of knowledge base, which was produced using 23 fuzzy rules, is represented in Table 1. It is completed using expert rules from (5), some of them being generated in the previous section. The two fuzzy inputs characterizing the mean values, *Mean-NS* and *Mean-TF*, divide the permitted area in *ℜ* ^2^, resulting in 9 different zones, similar to those presented in Figure 4.

The membership functions of the input variables are listed with their simplified names in bold-type in the table head (see Table 1). For output variable, the membership functions in the table cells are represented with their abbreviations, specified above. For the sake of clarity, the 9 zones in the permitted area are also highlighted.

In general, the output of fuzzy system is moving on output surface generated by inference process based on the input combinations. The system behaviour can be observed on this surface. If the input number, denoted *n*, is high, then the output surface has also high dimension. In this case, the system behaviour can be studied on projections of output surface in 2-dimensional input subspace with all other inputs considered as constants. Using combinations of *n* objects taken *k* at a time, denoted *C*_*n*_^*k*^, there are *C*_*n*_^2^ projection classes of output surface. In each class, the constant fuzzy inputs can have different values.

For MOP-FDS with 4 fuzzy inputs, there are 6 projection classes of the output surface. Due to their meanings, the two fuzzy inputs characterizing the mean values, *Mean-NS,* and *Mean-TF*, are chosen as constant inputs. Hence, only this class is considered in the paper, in which the projections of output surface depend on *NS* and *TF* fuzzy variables. In the class, 9 different projections can be generated, corresponding to zones shown in Figure 4.

## 5. Simulation Results

To illustrate the advantages of fuzzy logic in predictive diagnosis and early detection of the fault tendencies, a large input data set is generated. It contains hourly records of operating points, (*x*_1_ = *N*_*S*_,  *x*_2_ = *T*_*F*_) ∈ *ℜ* ^2^, for a time horizon of 5 years. During this time, the medium- and long-term mean values have variations similar to those shown in Figure 3. As a result, a mean value trajectory appears as combination of seasonal movements with long-term shifting. The short-term components are considered as random variables with normal distribution and zero mean. 

**Figure 8:**
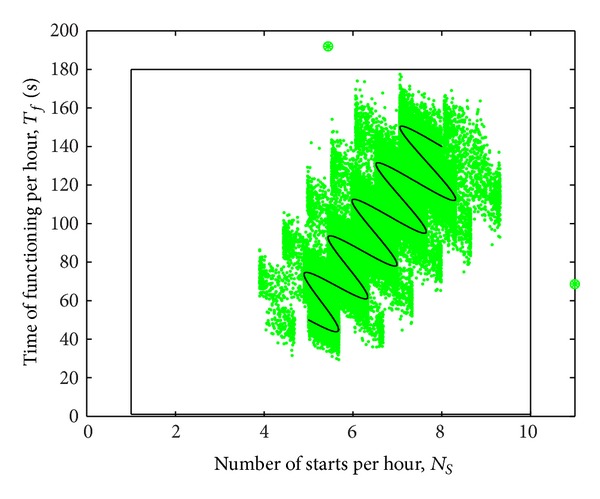
Input data set for 5 year-time horizon.

The data set contains 43800 points in *ℜ* ^2^, which are illustrated with grey color in Figure 8. The resulting variation of mean values is represented with a dark continuous line. Two points are placed intentionally outside the permitted area, being represented with both “o” and “∗” marks.

The two points represent values exceeding the range for either variable, *N*_*S*_ and *T*_*F*_. The maximum values are exceeded at two particular moments of time, at the 10000th sample and 15000th sample, respectively.

**Figure 9:**
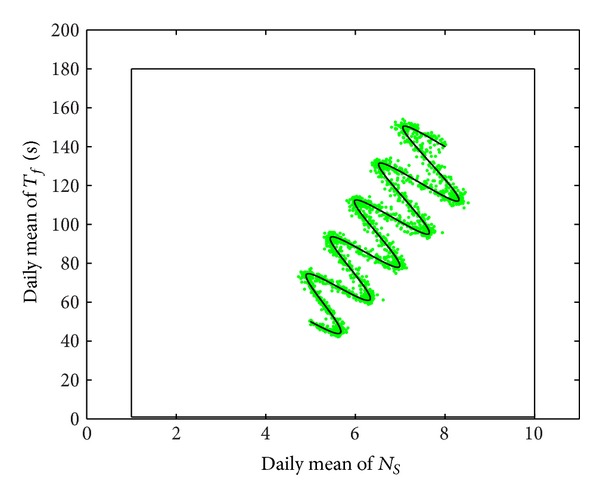
Daily averages in the input data set.

To verify the distribution around the mean values, the daily averages are computed in the data set and shown with grey color in Figure 9. It can be observed that the daily-mean points follow the variation of the mean values.

**Figure 10:**
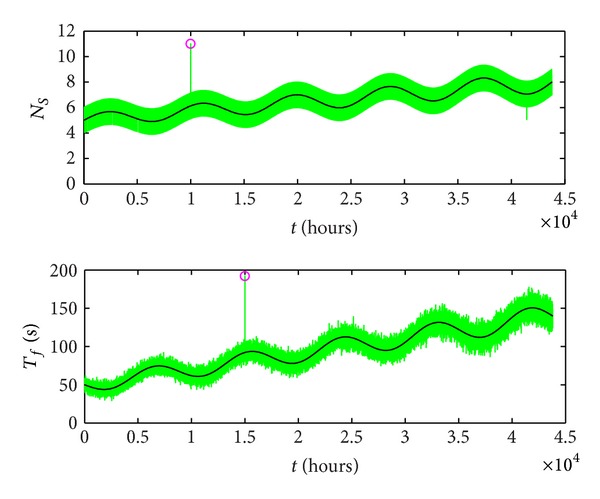
Coordinates of operating points.

The coordinates of operating points, (*x*_1_ = *N*_*S*_,  *x*_2_ = *T*_*F*_) ∈ *ℜ* ^2^, are functions of time with hourly records, being represented in Figure 10.

The two points placed outside the permitted area are represented with “o” marks. It can be observed that the second coordinate, time of functioning per hour, is noisier because it contains real values, expressed with 2 digits after the decimal point. The first coordinate, number of starts per hour, is an integer.

Several fuzzy diagnostic systems were used in simulations, with different characteristics of knowledge base and membership functions. Comparative results from 6 fuzzy systems, denoted MOP-FDSi, *i* = 1,…, 6, are presented in the paper. These 6 fuzzy systems were generated using 3 different rule bases and 2 sets of membership functions.

MOP-FDS1 has the membership functions and rule base presented in Section 4. For predictive diagnosis with the given data set, the most useful projections of output surface are those around the upper-right corner in the permitted area.

**Figure 11:**
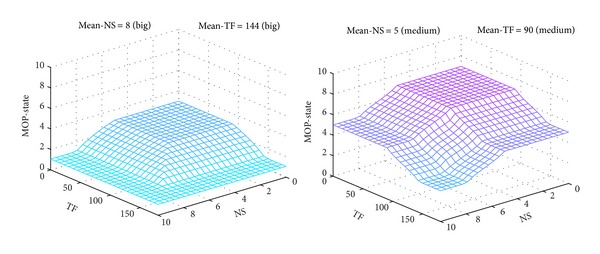
MOP-FDS1 output surface for pairs (*Big*, *Big*) and (*Medium*, *Medium*).

The projections of output surface of MOP-FDS1, corresponding to pairs of constant inputs (*Mean-NS* = *Big*, *Mean-TF* = *Big*) and (*Mean-NS* = *Medium*, *Mean-TF* = *Medium*), respectively, are shown in Figure 11.

The projection for the pair (*Mean-NS* = *Small*, *Mean-TF* = *Small*) has similar shape as the one for pair (*Medium*, *Medium*), but shifted to higher values of *MOP*-*State*.

**Figure 12:**
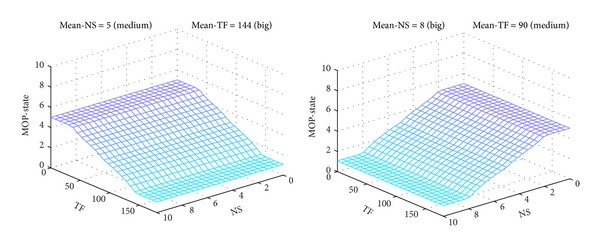
MOP-FDS1 output surface for pairs (*Medium*, *Big*) and (*Big*, *Medium*).

The MOP-FDS1 projections of output surface, corresponding to pairs of constant inputs (*Mean-NS* = *Medium*, *Mean-TF* = *Big*) and (*Mean-NS* = *Big*, *Mean-TF* = *Medium*), respectively, are illustrated in Figure 12. The rest of the 4 projections have similar shapes, but they shifted to different values of *MOP-State*, accordingly with rule base given in Table 1.

The odd numbered fuzzy systems, MOP-FDSi with *i* = 1,3, and 5, have different rule bases, but the same membership functions, presented in Figures 6 and 7.

The even numbered systems, with *i* = 2,4, and 6, have all the same membership functions, which are different from those presented in Figures 6 and 7, with more narrow intersection areas. Their rule bases are the same as their corresponding paired odd numbered systems (e.g., MOP-FDS1 and MOP-FDS2 have the same rule base, but different membership functions).

**Figure 13:**
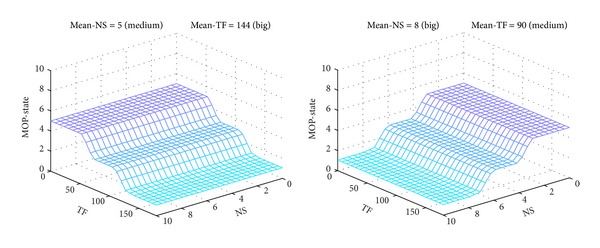
MOP-FDS2 output surface for pairs (*Medium*, *Big*) and (*Big*, *Medium*).

If the membership functions are modified to more narrow intersection areas, the output surface changes to a more rough shape. For example, the MOP-FDS2 projections of output surface, corresponding to pairs of constant inputs (*Mean-NS* = *Medium*, *Mean-TF* = *Big*) and (*Mean-NS* = *Big*, *Mean-TF* = *Medium*), respectively, are represented in Figure 13. The shapes illustrated in Figure 12 are smoother.

**Figure 14:**
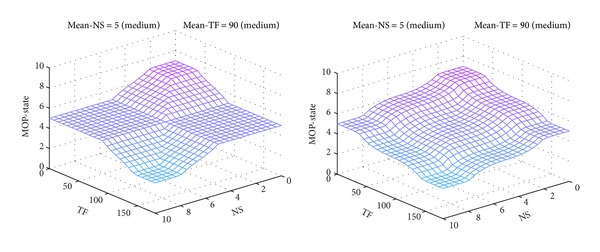
Output surface of MOP-FDS3 and MOP-FDS5 for pair (*Medium*, *Medium*).

If the rule base is changed with more complex rules, the output surface changes accordingly. For example, the projections of output surface, corresponding to the pair of constant inputs (*Mean-NS* = *Medium*, *Mean-TF* = *Medium*) for MOP-FDS3 and MOP-FDS5, respectively, are shown in Figure 14. It can be observed that they are different from the same projection shown in Figure 11. The smoothest surface is obtained for MOP-FDS5.

The output performance of MOP-FDSi, *i* = 1,…, 6 is represented in Table 2. 

Two performance criteria were used. The first one is the mean squared error (mse) of fuzzy output (*MOP-State*) compared with a theoretical linear decreasing tendency of MOP functioning state.

The second performance criterion is the total functioning time (*T*_max⁡_) while the fuzzy output remains above a theoretical alarm value, which was chosen at *MOP-State* = 2.

It can be observed that all the even numbered fuzzy systems have worse performance than their paired systems with the same rule base but with membership functions presented in Figures 6 and 7. The best performance was obtained for MOP-FDS3, with mse = 0.4565.

The total functioning time (*T*_max⁡_) has comparable values for all fuzzy diagnostic systems (little above 4 years). Again, MOP-FDS3 has the fastest predictive diagnostic response of MOP state.

**Figure 15:**
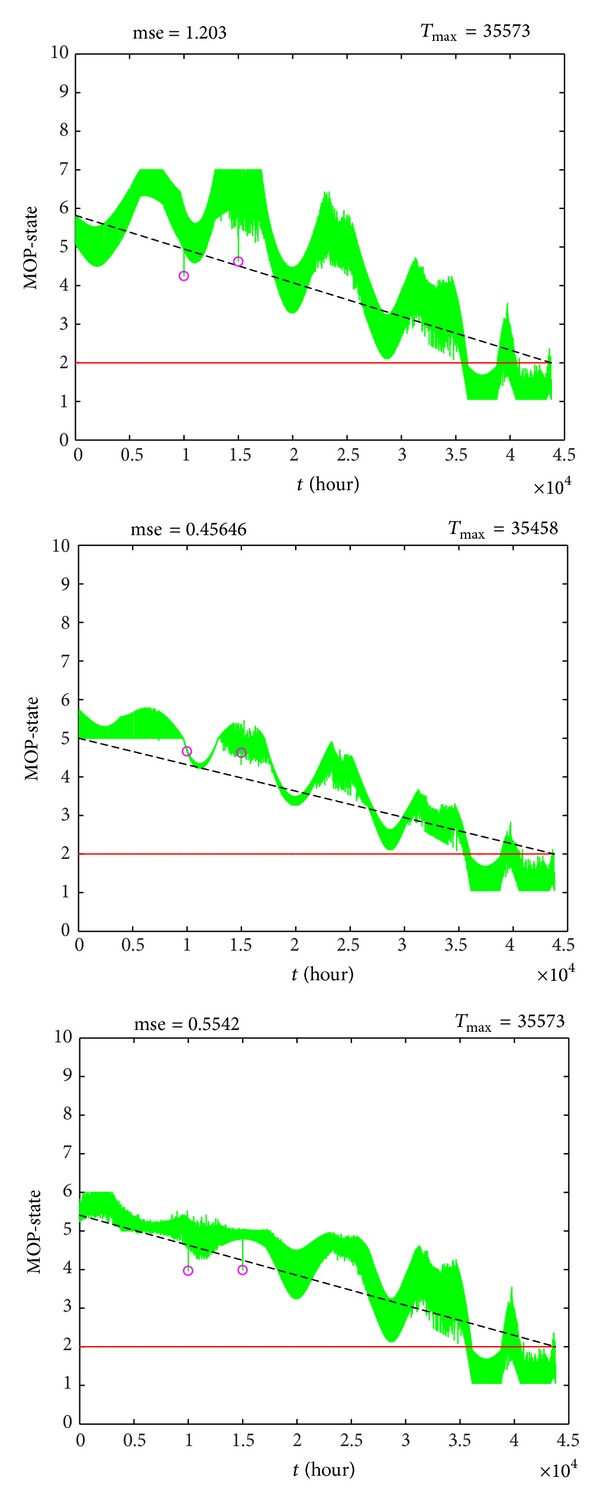
Fuzzy outputs of odd MOP-FDSi.

The three outputs of odd numbered fuzzy systems are shown with grey color in Figure 15. As expected, the outputs decrease in time, from a state around *Normal* to the *Very-Bad* state. As it can be observed, the dispersion of the points is larger for MOP-FDS1.

When the two points cross the border of permitted area, the outputs have small variations, shown with “o” mark, but they do not make major changes in MOP state. By contrary, using a classic crisp system, each of these points would be interpreted as a fault, activating an error and stopping the MOP.

At *T*_max⁡_ time sample (little over 4 years), the fuzzy outputs decrease under alarm line, represented with horizontal continuous line. At this moment, the MOP state is already *Bad*, even it is not defective yet. The MOP functioning is not reliable, and maintenance process should be scheduled.

## 6. Conclusions

Fuzzy rules are generated to characterize the functioning state of MOP-type drive mechanisms. Several fuzzy systems are proposed for online predictive diagnosis. The fuzzy output is not perturbed by operating points crossing the border of permitted area for short time periods. The fuzzy systems work well, making an early detection of the fault tendencies of MOP mechanism.

## Figures and Tables

**Table 1:** Rule base example of FDS.

Mean-NS	NS	Mean-TF								
		Small (S)			Medium (M)			Big (B)		
		TF								
		S	M	B	S	M	B	S	M	B
S	S	VG	VG	G	G	N	B	N	B	VB
	M	VG	VG	G	G	N	B	N	B	VB
	B	G	G	N	G	N	B	N	B	VB

M	S	G	G	G	G	G	N	N	B	VB
	M	N	N	N	G	G	N	N	B	VB
	B	B	B	B	N	N	B	N	B	VB

B	S	N	N	N	N	N	N	B	B	VB
	M	B	B	B	B	B	B	B	B	VB
	B	VB	VB	VB	VB	VB	VB	VB	VB	VB

**Table 2:** Output performance of MOP-FDS.

Fuzzy diagnostic system	FDS output performance	
	mse	*T*_max⁡_ (hours)
MOP-FDS1	1.2030	35573
MOP-FDS2	1.9371	35573
MOP-FDS3	0.4565	35458
MOP-FDS4	0.5378	35558
MOP-FDS5	0.5542	35573
MOP-FDS6	0.7712	35573

## References

[1] Ghosh A, Ledwich G (2002). *Power Quality Enhancement Using Custom Power Devices*.

[2] Nguyen TC, Chan S, Bailey R, Nguyen T (2002). Auto-check circuit breaker interrupting capabilities. *IEEE Computer Applications in Power*.

[3] Sweetser C, Bergman WJ, Montillet G (2002). Strategies for selecting monitoring of circuit breakers. *IEEE Transactions on Power Delivery*.

[4] Eissa MM (2002). Automating motor-operated air-breaker switches. *IEEE Computer Applications in Power*.

[5] Miciu I, Hartescu F (2009). Monitoring system for co-generative power plants. *Studies in Informatics and Control*.

[6] Dupraz JP, Schiemann A, Montillet GF Design objectives of new digital control and monitoring of high voltage circuit breakers.

[7] M’halla A, Craye E, Dutilleul SC, Benrejeb M (2010). Monitoring of a milk manufacturing workshop using chronicle and fault tree approaches. *Studies in Informatics and Control*.

[8] Dewulf JA, Jung T, Dupraz JP, Montillet GF A development and application of circuit breakers diagnostic and monitoring.

[9] Monticelli A (2000). Electric power system state estimation. *Proceedings of the IEEE*.

[10] Allan R, Billinton R (2000). Probabilistic assessment of power systems. *Proceedings of the IEEE*.

[11] Manea I, Ionescu E, Irimia D Modern methods to diagnose the oleo-pneumatic operating mechanisms type MOP.

[12] Stanek M, Fröhlich K (2000). Model-aided diagnosis—a new method for online condition assessment of high voltage circuit breakers. *IEEE Transactions on Power Delivery*.

[13] Hong-Chan C (2003). Fault section diagnosis of power system using fuzzy logic. *IEEE Transactions on Power Systems*.

[14] Min SW, Sohn JM, Park JK, Kim KH (2004). Adaptive fault section estimation using matrix representation with fuzzy relations. *IEEE Transactions on Power Systems*.

[15] Sun J, Qin SY, Song YH (2004). Fault diagnosis of electric power systems based on fuzzy petri nets. *IEEE Transactions on Power Systems*.

[16] Tan JC, Crossley PA, McLaren PG Fuzzy expert system for on-line fault diagnosis on a transmission network.

[17] Ibrahim WRA, Morcos MM (2001). Adaptive fuzzy technique for learning power-quality signature waveforms. *IEEE Power Engineering Review*.

[18] Shoureshi R, Norick T, Linder D, Work J, Kaptain P Sensor fusion and complex data analysis for predictive maintenance.

[19] Shoureshi R, Norick T, Permana V, Work J Advanced sensor and diagnostic technologies for development of intelligent substations.

